# Risk Factor Analysis of Death in Patients With Hepatic Cellular Carcinoma After Radical Operation: A Consecutive Cohort of 433 Patients

**DOI:** 10.1002/hsr2.71428

**Published:** 2025-10-30

**Authors:** Zhengyang He, Wenfeng Lu, Dongze Qiu, Weimin She

**Affiliations:** ^1^ Department of Integrative Medicine Shanghai Geriatrics Medical Center Shanghai China; ^2^ Department of Integrative Medicine, Zhongshan Hospital Fudan University Shanghai China; ^3^ Department of Gastroenterology, Zhongshan Hospital Fudan University Shanghai China

**Keywords:** cohort study, hepatic cellular carcinoma, radical operation, risk factors, survival

## Abstract

**Objective:**

No matter what kind of radical operation, the recurrence and metastasis of tumor will seriously affect the postoperative long‐term effect, and hinder the survival of hepatic cellular carcinoma (HCC) patients. This study aims to explore the relevant risk factors through multivariable analysis, to provide basis for the screening of prognostic factors of HCC and the prevention of tumor related events.

**Methods:**

433 patients with HCC after radical operation were involved in this study. The general clinical data, pathological data and laboratory indicators of patients were analyzed through univariate and multivariable analysis. Finally, the independent risk factors of disease free survival (DFS) and overall survival (OS) in patients were screened out.

**Results:**

The 1‐, 3‐, and 5‐year DFS rates were 70.67%, 49.65% and 40.65%, while the 1‐, 3‐, and 5‐year OS rates were 90.30%, 79.68% and 70.67%, respectively. Multivariable analysis showed that tumor number, microvascular invasion, thickness of resection margin, AFP, AST and GGT were the independent risk factors of DFS, while PS score, tumor number, maximal tumor size, ES classification, microvascular invasion, AST and GGT were the independent risk factors of OS (*p* < 0.05).

**Conclusion:**

Tumor number, microvascular invasion, AST and GGT are correlated with both shorter DFS and OS, which means they can be considered as predictors of the prognosis and recurrence in patients with HCC after radical operation. Patients with these risk factors should be screened in time, which is of great significance to prevent tumor related events and improve survival.

**Abbreviations**
AFP: alpha fetoprotein
AST: aspartate aminotransferase
DFS: disease free survival
ES: Edmond‐Steiner
GGT: γ
  glutamyl transpeptidase
HCC: hepatic cellular carcinoma
OS: overall survival
PHC: primary hepatic carcinoma
PS: performance status
RFA: radio frequency ablation
TACE: transcatheter arterial chemoembolization
TNM: tumor‐node‐metastasis

## 1 Introduction

At present, primary hepatic carcinoma (PHC) ranks sixth in the global incidence of malignant tumor and it is the fourth leading cause of cancer‐related mortalities in the world. There are nearly 840,000 new‐onset PHC patients, while 780,000 people die of the disease worldwide every year [1]. PHC has the characteristics of insidious onset, rapid progress, high malignant degree and difficult to cure, which seriously endangers people′s lives and health. The main pathology of PHC includes hepatic cellular carcinoma (HCC), intraepithelial cholangiocarcinoma (ICC) and HCC‐ICC mixed type, while HCC is the most common type, accounting for more than 90% of PHC patients [2].

Nowadays, there are many options for the treatment of HCC. Surgical intervention, local ablation, transcatheter arterial chemoembolization (TACE), systematic treatment, traditional Chinese medicine and other treatments have developed rapidly in recent years. Due to the lack of liver donors, radical hepatectomy is still the most effective treatment for HCC patients [3]. However, even after radical operation at early stage, 60% of HCC patients have recurrence within 5 years, which ultimately affects the long‐term survival rate [4].

In addition to hepatectomy, the application of local ablation is also gradually increasing, among which radio frequency ablation (RFA) is considered to have curative effect on early HCC and is widely recommended by domestic and foreign guidelines [5, 6]. The promotion of RFA brings new modes and choices for the radical treatment of HCC. Ablation is advanced in easy operation and minimal trauma compared with hepatectomy, however, it still has the problem of relatively high recurrence rate after operation. No matter what kind of radical operation, the recurrence and metastasis of tumor will seriously affect the postoperative long‐term effect, and hinder the survival of HCC patients. Prolonging the survival time of patients after radical operation and reducing recurrence and metastasis are still the difficulties that need to be overcome urgently.

Therefore, analyzing the risk factors of death in HCC patients after radical operation has important clinical value for developing individual strategies to prevent recurrence, which will help to improve the prognosis of HCC patients. This study aims to explore the relevant risk factors through multivariable analysis, to provide basis for the screening of prognostic factors of HCC and the establishment of individualized treatment system.

## 2 Materials and Methods

### 2.1 Patient Case Selection

We obtained ethical approval from our ethics committee to perform this study (Approval No.: B2021‐806R) and written informed consent was obtained from all research subjects for participation and publishing of the data. Patients treated for HCC after radical operation in database records of our hospital were retrospectively analyzed from Jan 1, 2016, to Jan 31, 2021 (updated medical record system was used since Jan 1, 2016).

Inclusion criteria were as follows: (1) patients diagnosed as HCC by pathology; (2) radical operation was the first treatment method conducted between Jan 2011 and Jan 2016 after diagnosis (resection or ablation); (3) score 0–2 of the performance status (PS) based on standard of Eastern Cooperative Oncology Group [7], grade A or B of Child‐Pugh classification [8, 9]; (3) clinical stage I–III based on tumor‐node‐metastasis (TNM) staging [10]; (4) postoperative treatment mainly included general symptomatic treatment, TACE and systematic treatment (including targeted therapy, radiotherapy and chemotherapy).

Exclusion criteria including: (1) patients with serious adverse reactions during postoperative treatment; (2) patients with vascular invasion or extrahepatic metastasis; (3) patients with serious basic liver or other systematic disease (including other malignant tumors); (4) patients with incomplete original case data.

### 2.2 Observation Indicators

The general clinical data were collected respectively, including age, sex, hepatitis, ascites, smoking, drinking, family history of liver cancer, tumor size and number, PS score, Child‐Pugh classification, and surgical procedure (open or laparoscopic, combined with TACE/RFA or not).

The pathology report included Edmond‐Steiner (ES) classification [11], capsule formation, microvascular invasion, satellite nodule, thickness of resection margin, cirrhosis and TNM staging.

In addition, the laboratory indicators of patients were compared, including alpha fetoprotein (AFP), total bilirubin (TBIL), albumin (ALB), prealbumin (PAB), alanine aminotransferase (ALT), aspartate aminotransferase (AST), γ‐glutamyl transpeptidase (GGT), and blood platelet (PLT).

The outcome indicators were disease free survival (DFS) and overall survival (OS).

### 2.3 Statistical Analysis

Statistical Packages of Social Sciences (SPSS) software (version 22.0) was used to analyze the collected data. The DFS and OS were analyzed using the Kaplan–Meier method, and the survival curves were compared using the Log‐rank test. The Cox′s proportional hazards model was used for multivariable survival analysis. Through comparing the differences between the survival curve predicted by the Cox model and the actual survival curve obtained by the Kaplan‐Meier method, it can be determined whether the proportional hazards assumption of the Cox model is valid. A *p*‐value of less than 0.05 was considered statistically significant.

## 3 Result

**Figure 1:**
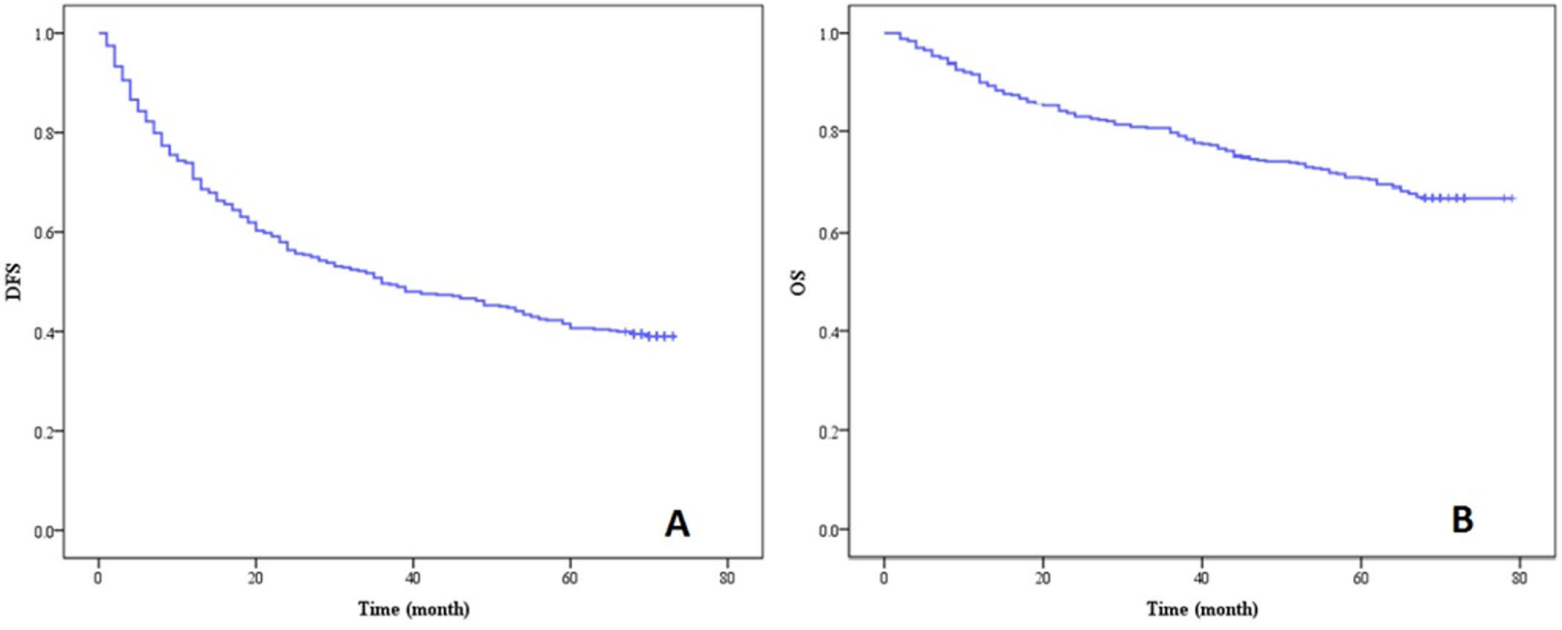
(A) DFS and (B) OS curve of patients with HCC after radical operation.

A total of 433 consecutive cases were involved in the current study, including 373 males and 60 females with a mean age of 56.19 ± 9.27 years (ranges from 24 to 87 years). Based on Log‐rank test, the DFS and OS curve of patients were shown in Figure 1 (A‐B). The 1‐, 3‐, and 5‐year DFS rates were 70.67%, 49.65% and 40.65%, respectively, with a median DFS time of 36.00 (24.47–47.53) months, while the 1‐, 3‐, and 5‐year OS rates were 90.30%, 79.68% and 70.67%, respectively.

### 3.1 Univariate Analysis

#### 3.1.1 Clinical Data Variables

Based on Kaplan‐Meier method, the analysis showed that the PS score, tumor number, maximal tumor size and combination with TACE/RFA were closely related to both DFS and OS of patients, which had statistical significance (PS score, *p* = 0.002 for DFS and < 0.001 for OS; tumor number, *p* < 0.001 for DFS and < 0.001 for OS; maximal tumor size, *p* < 0.001 for DFS and < 0.001 for OS; combination with TACE/RFA, *p* = 0.004 for DFS and = 0.012 for OS). Other clinical data variables of patients had no inevitable correlation with either their DFS or OS (sex, *p* = 0.853 for DFS and = 0.152 for OS; age, *p* = 0.236 for DFS and = 0.759 for OS; Child‐Pugh Class, *p* = 0.564 for DFS and = 0.776 for OS; hepatitis, *p* = 0.799 for DFS and = 0.300 for OS; ascites, *p* = 0.091 for DFS and = 0.189 for OS; smoking, *p* = 0.810 for DFS and = 0.251 for OS; drinking, *p* = 0.607 for DFS and = 0.089 for OS; family history, *p* = 0.653 for DFS and = 0.090 for OS; surgical procedure, *p* = 0.298 for DFS and = 0.090 for OS) (Table 1).

**Table 1:** Univariate Analysis (Kaplan–Meier Method) of Patients′ Clinical Data for DFS and OS.

Variables	Number	DFS			*p*‐value	OS			*p*‐value
		1‐ year (%)	3‐ year (%)	5‐ year (%)		1‐ year (%)	3‐ year (%)	5‐ year (%)	
Sex									
Female	60	68.33	45.00	40.00	0.853	85.00	73.33	63.33	0.152
Male	373	71.05	50.40	40.75		91.15	80.70	71.85	
Age									
< 65	336	70.24	47.92	39.29	0.236	90.48	79.17	70.24	0.759
≥ 65	97	72.16	55.67	45.36		89.69	81.44	72.16	
PS score									
0	285	79.08	56.74	44.68	0.002*	93.97	86.88	77.66	< 0.001*
≥ 1	148	55.10	36.05	34.01		83.67	65.99	57.82	
Child‐Pugh Class									
A	429	70.63	49.42	40.56	0.564	90.21	79.72	70.63	0.776
B	4	75.00	75.00	50.00		100.00	75.00	75.00	
Hepatitis									
Negative	43	60.47	53.49	48.84	0.799	88.37	62.79	60.47	0.300
HBV	384	71.88	49.22	39.58		90.36	81.25	71.88	
HCV	6	66.67	50.00	50.00		100.00	100.00	66.67	
Ascites									
Absent	425	70.82	50.12	41.18	0.091	90.59	80.00	71.06	0.189
present	8	62.50	25.00	12.50		75.00	62.50	50.00	
Smoking									
no	206	68.93	47.57	41.75	0.810	87.86	77.18	67.48	0.251
yes	227	72.25	51.54	39.65		92.51	81.94	73.57	
Drinking									
no	221	70.59	47.51	39.37	0.607	88.24	76.47	66.97	0.089
yes	212	70.75	51.89	41.98		92.45	83.02	74.53	
Family history									
no	350	70.29	49.71	41.71	0.653	90.00	78.29	68.57	0.090
yes	83	72.29	49.40	36.14		91.57	85.54	79.52	
Tumor number									
1	323	75.85	55.73	46.75	< 0.001*	92.26	83.59	75.85	< 0.001*
2–3	88	59.09	35.23	25.00		90.91	72.73	59.09	
≥ 4	22	40.91	18.18	13.64		59.09	50.00	40.91	
Maximal tumor size									
≤ 3 cm	168	83.33	66.07	52.98	< 0.001*	95.83	89.88	85.12	< 0.001*
3–5 cm	107	71.03	46.73	36.45		96.26	85.05	68.22	
＞5 cm	158	56.96	34.18	30.38		80.38	65.19	56.96	
Surgical procedure									
Open	399	69.92	49.12	40.10	0.298	89.72	78.20	69.17	0.090
Laparoscopy	34	79.41	55.88	47.06		97.06	97.06	88.24	
TACE/RFA									
NO	379	72.82	52.24	42.48	0.004*	90.77	81.00	72.30	0.012*
yes	54	55.56	31.48	27.78		87.04	70.37	59.26	

Abbreviations: DFS, disease free survival; HBV, Hepatitis B Virus; HCV, Hepatitis C Virus; OS, overall survival; PS, performance status; RFA, radio frequency ablation; TACE, transcatheter arterial chemoembolization.

* The factor has statistical significance to DFS or OS.

#### 3.1.2 Pathological Data Variables

Based on Kaplan–Meier method, the analysis showed that the ES classification, capsule formation, microvascular invasion, satellite nodule, thickness of resection margin and TNM staging were closely related to the DFS of patients (ES classification, *p* < 0.001; capsule formation, *p* = 0.049; microvascular invasion, *p* < 0.001; satellite nodule, *p* = 0.005; thickness of resection margin, *p* = 0.045; TNM staging, *p* < 0.001), while the ES classification, microvascular invasion, satellite nodule and TNM staging were closely related to the OS of patients, which had statistical significance (ES classification, *p* < 0.001; microvascular invasion, *p* < 0.001; satellite nodule, *p* < 0.001; TNM staging, *p* < 0.001). Other pathological data variables of patients had no inevitable correlation with either DFS or OS (capsule formation, *p* = 0.143 for OS; thickness of resection margin, *p* = 0.348 for OS; cirrhosis, *p* = 0.556 for DFS and = 0.119 for OS) (Table 2).

**Table 2:** Univariate Analysis (Kaplan‐Meier Method) of Patients′ Pathological Data for DFS and OS.

Variables	Number	DFS			*p* value	OS			*p* value
		1‐ year (%)	3‐ year (%)	5‐ year (%)		1‐ year (%)	3‐ year (%)	5‐ year (%)	
ES classification									
I–II	256	77.34	55.47	45.70	< 0.001*	94.14	85.16	76.95	< 0.001*
III	177	61.02	41.24	33.33		84.75	71.75	61.58	
Capsule formation									
Absent	153	62.75	43.14	36.60	0.049*	86.27	73.86	66.67	0.143
present	280	75.00	53.21	42.86		92.50	82.86	72.86	
Microvascular invasion									
Absent	266	78.20	58.27	47.37	< 0.001*	95.49	87.97	77.07	< 0.001*
present	167	58.68	35.93	29.94		82.04	66.47	60.48	
Satellite nodule									
Absent	368	73.91	51.90	42.39	0.005*	92.39	83.42	74.18	< 0.001*
present	65	52.31	36.92	30.77		78.46	58.46	50.77	
Thickness of resection margin									
< 1 cm	260	66.54	45.77	38.08	0.045*	89.23	77.69	69.23	0.348
≥ 1 cm	173	76.88	55.49	44.51		91.91	82.66	72.83	
Cirrhosis									
Absent	192	69.27	46.88	39.58	0.556	86.98	75.52	67.19	0.119
present	241	71.78	51.87	41.49		92.95	82.99	73.44	
TNM staging									
IA	70	84.29	64.29	51.43	< 0.001*	97.14	91.43	84.29	< 0.001*
IB	158	77.85	56.96	48.73		95.57	86.71	77.22	
II	143	67.13	45.45	35.66		87.41	77.62	67.83	
IIIA	62	45.16	24.19	19.35		75.81	53.23	45.16	

Abbreviations: ChatGPT said:DFS, disease free survival; ES, Edmond–Steiner; OS, overall survival; TNM, tumor‐node‐metastasis.

* The factor has statistical significance to DFS or OS.

#### 3.1.3 Laboratory Indicators

Based on Kaplan‐Meier method, the analysis showed that the AFP, PAB, AST and GGT were closely related to both DFS and OS of patients, which had statistical significance (AFP, *p* < 0.001 for DFS and < 0.001 for OS; PAB, *p* = 0.028 for DFS and < 0.001 for OS; AST, *p* < 0.001 for DFS and < 0.001 for OS; GGT, *p* = 0.004 for DFS and = 0.012 for OS). Other laboratory indicators of patients had no inevitable correlation with either their DFS or OS (TBIL, *p* = 0.453 for DFS and = 0.737 for OS; ALB, *p* = 0.062 for DFS and = 0.219 for OS; ALT, *p* = 0.238 for DFS and = 0.154 for OS; PLT, *p* = 0.257 for DFS and = 0.479 for OS) (Table 3).

**Table 3:** Univariate Analysis (Kaplan–Meier Method) of Patients' Laboratory Indicators for DFS and OS.

Variables	Number	DFS			*p*‐value	OS			*p*‐value
		1 year (%)	2 year (%)	5 year (%)		1 year (%)	3 year (%)	5 year (%)	
AFP									
< 400	316	78.48	57.28	45.89	< 0.001*	92.09	83.86	75.95	< 0.001*
≥ 400	117	49.57	29.06	26.50		85.47	68.38	56.41	
TBIL									
< 17.1	362	70.44	48.34	39.50	0.453	90.06	79.28	70.72	0.737
17.1–34.2	65	73.85	58.46	47.69		90.77	81.54	69.23	
> 34.2	6	50.00	33.33	33.33		100.00	83.33	83.33	
ALB									
< 35	28	53.57	42.86	25.00	0.062	82.14	75.00	67.86	0.219
≥ 35	405	71.85	50.12	41.73		90.86	80.00	70.86	
PAB									
< 200	145	65.52	43.45	33.10	0.028*	82.07	70.34	60.00	< 0.001*
≥ 200	288	73.26	52.78	44.44		94.44	84.38	76.04	
ALT									
< 40	284	73.94	51.41	41.55	0.238	90.49	80.99	72.89	0.154
≥ 40	149	64.43	46.31	38.93		89.93	77.18	66.44	
AST									
< 40	290	78.97	57.59	46.55	< 0.001*	93.45	87.59	79.66	< 0.001*
≥ 40	143	53.85	33.57	28.67		83.92	63.64	52.45	
GGT									
< 60	230	81.74	58.70	48.70	< 0.001*	95.22	90.43	82.17	< 0.001*
≥ 60	203	58.13	39.41	31.53		84.73	67.49	57.64	
PLT									
< 100	63	69.84	44.44	31.75	0.257	87.30	73.02	65.08	0.479
≥ 100	370	70.81	50.54	42.16		90.81	80.81	71.62	

Abbreviations: AFP, alpha fetoprotein; ALB, albumin; ALT, alanine aminotransferase; AST, aspartate aminotransferase; DFS, disease free survival; GGT, γ‐glutamyl transpeptidase; OS, overall survival; PAB, prealbumin; PLT, blood platelet; TBIL, total bilirubin.

* The factor has statistical significance to BM.

### 3.2 Multivariable Analysis

#### 3.2.1 Risk Factors of DFS

**Figure 2:**
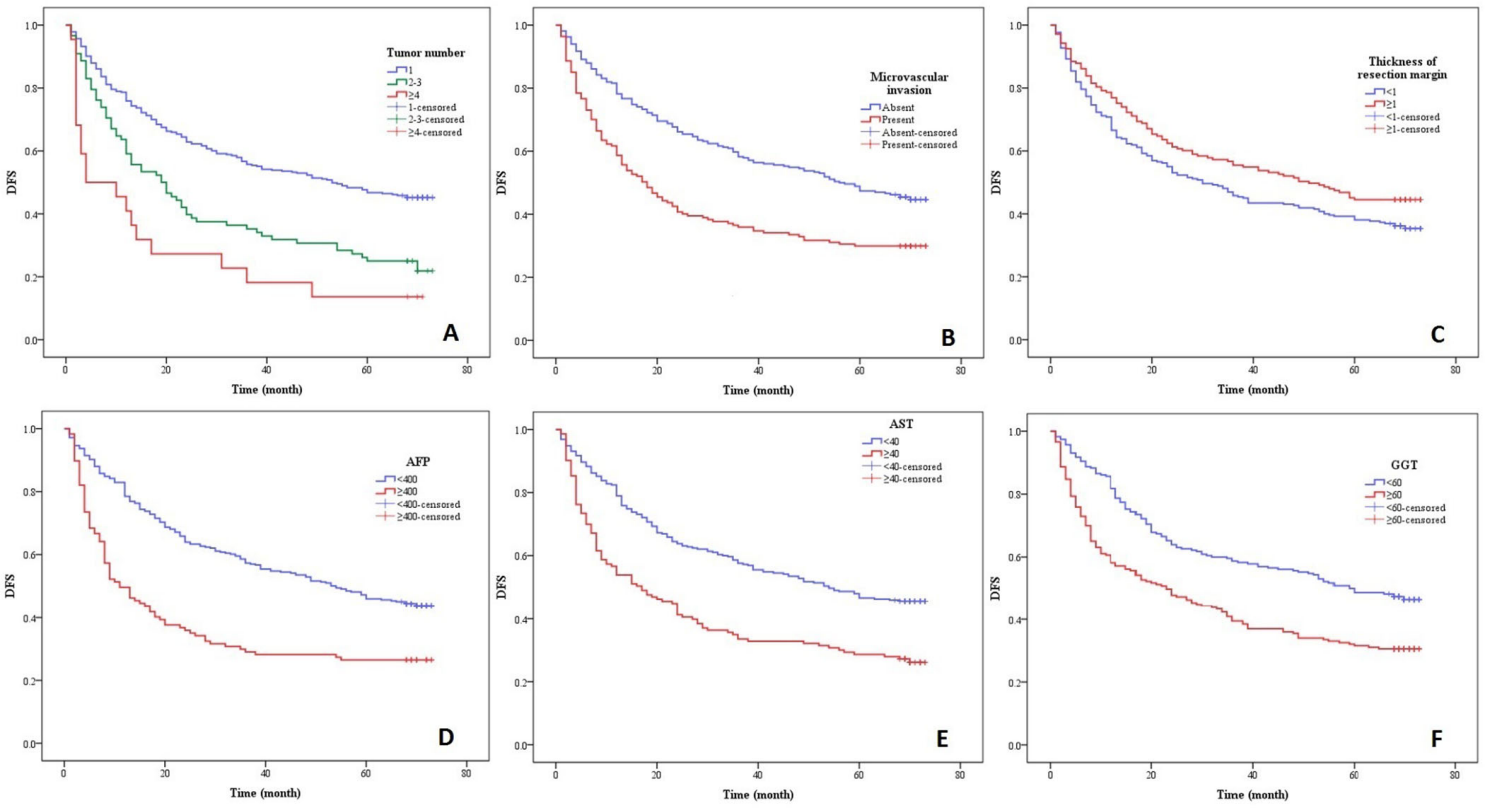
DFS in patients with HCC after radical operation according to (A) tumor number, (B) microvascular invasion, (C) thickness of resection margin, (D) AFP, (E) AST and (F) GGT.

The DFS related factors in the above univariate analysis were included in the Cox regression multivariable analysis. The results showed that the tumor number 2–3 (β = 0.475, 95%CI = 1.200–2.154), tumor number ≥ 4 (β = 1.251, 95%CI = 2.136–5.715), presence of microvascular invasion (β = 0.427, 95%CI = 1.189–1.975), thickness of resection margin ≥ 1 (β = −0.368, 95%CI = 0.534–0.897), AFP ≥ 400 (β = 0.480, 95%CI = 1.229–2.125), AST ≥ 40 (β = 0.446, 95%CI = 1.196–2.041) and GGT ≥ 60 (β = 0.291, 95%CI = 1.031–1.735) were significantly correlated with the shorter DFS (*p* < 0.05). Meanwhile, the larger tumor number with higher β represented greater correlation (Table 4). Therefore, the tumor number, microvascular invasion, thickness of resection margin, AFP, AST and GGT were the significant independent risk factors of DFS, while the PS score, maximal tumor size, combination with TACE/RFA, ES classification, capsule formation, satellite nodule, TNM staging and PAB were the risk factors without significance. The DFS curve of each factor was shown in Figure 2 (A–F).

**Table 4:** Multivariable Analysis (Cox Regression) of Risk Factors for DFS.

Variables	β	S.E.	Wald	*p*‐value	HR	95%CI	
PS score							
0	*Reference*						
≥ 1	0.127	0.154	2.628	0.137	0.749	0.583–1.106	
Tumor number			29.985	<0.001*			
1	*Reference*						
2–3	0.475	0.149	10.134	0.001*	1.608	1.200–2.154	
≥ 4	1.251	0.251	24.839	<0.001*	3.494	2.136–5.715	
Maximal tumor size			4.515	0.118			
≤ 3 cm	*Reference*						
3–5 cm	0.211	0.236	3.054	0.165	0.948	1.932–3.215	
> 5 cm	0.186	0.207	2.472	0.094	1.026	1.074–2.613	
TACE/RFA							
No	*Reference*						
Yes	0.149	0.183	2.436	0.205	0.861	0.973–1.415	
ES classification							
I–II	*Reference*						
III	0.206	0.195	3.019	0.087	1.374	1.648–2.563	
Capsule formation							
Absent	*Reference*						
Present	0.167	0.228	3.831	0.142	0.957	0.845–1.621
Microvascular invasion						
Absent	*Reference*					
Present	0.427	0.130	10.860	0.001*	1.532	1.189–1.975
Satellite nodule						
Absent	*Reference*					
Present	0.301	0.217	4.254	0.102	1.185	1.437–2.092
Thickness of resection margin						
< 1	*Reference*					
≥ 1	−0.368	0.133	7.710	0.005*	0.692	0.534–0.897
TNM staging			6.163	0.102		
IA	*Reference*					
IB	0.248	0.273	3.751	0.146	0.872	1.053–1.465
II	0.250	0.197	2.826	0.113	2.084	1.247–1.819
IIIA	0.316	0.246	3.419	0.075	1.653	2.015–3.627
AFP						
< 400	*Reference*					
≥ 400	0.480	0.140	11.825	0.001*	1.616	1.229–2.125
PAB						
< 80	*Reference*					
≥ 80	0.207	0.231	1.934	0.185	0.926	1.270–1.993
AST						
< 40	*Reference*					
≥ 40	0.446	0.136	10.719	0.001*	1.563	1.196–2.041
GGT						
< 60	*Reference*					
≥ 60	0.291	0.133	4.779	0.029*	1.337	1.031–1.735

Abbreviations: AFP, alpha fetoprotein; AST, aspartate aminotransferase; DFS, disease free survival; ES, Edmond‐Steiner; GGT, γ‐glutamyl transpeptidase; PAB, prealbumin; PS, performance status; TACE/RFA, transcatheter arterial chemoembolization/radio frequency ablation.

* The factor has statistical significance to DFS.

#### 3.2.2 Risk Factors of OS

**Figure 3:**
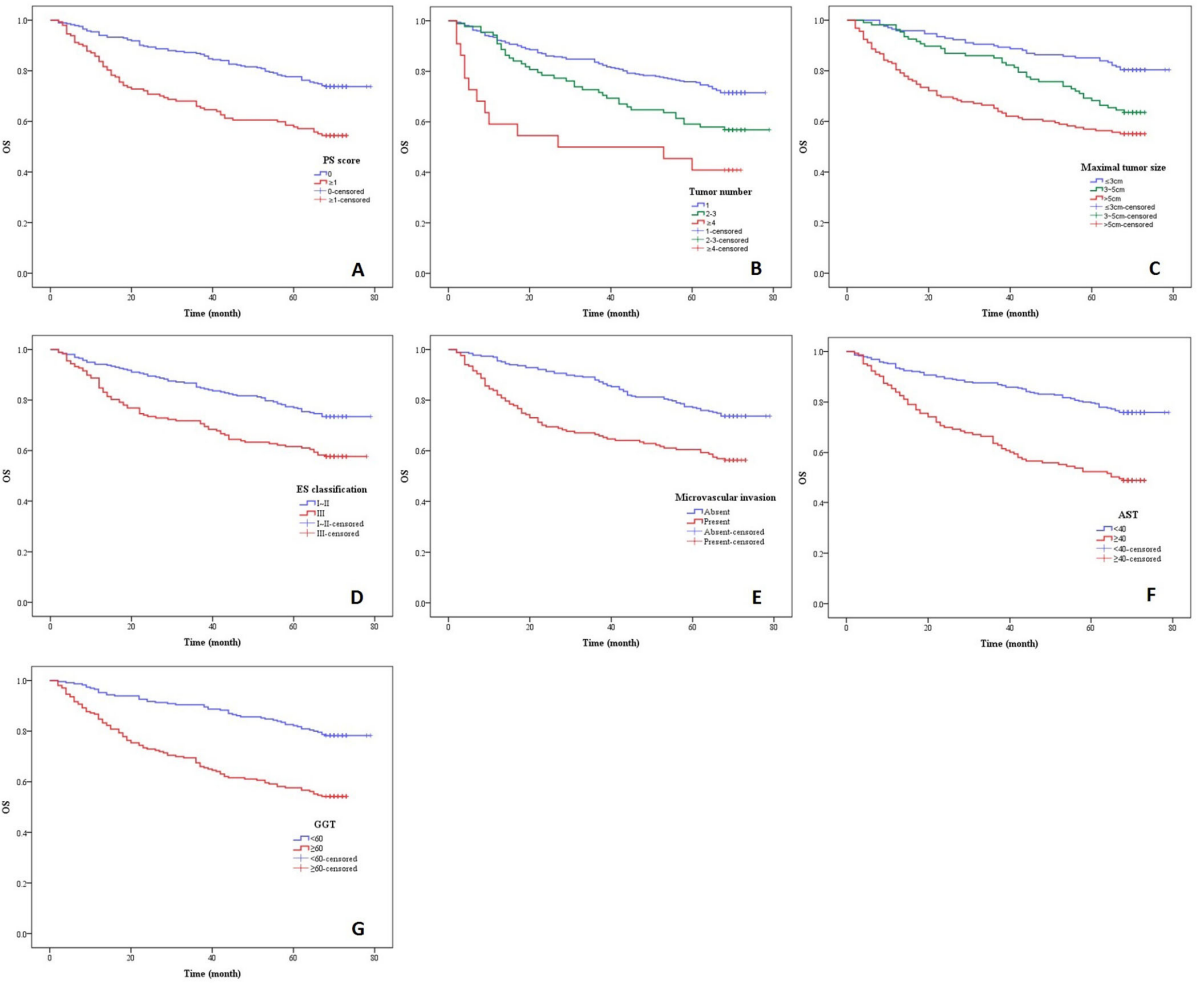
OS in patients with HCC after radical operation according to (A) PS score, (B) tumor number, (C) maximal tumor size, (D) ES classification, (E) microvascular invasion, (F) AST and (G) GGT.

The OS related factors in the above univariate analysis were included in the Cox regression multivariable analysis. The results showed that the PS score ≥ 1 (β = 0.374, 95%CI = 1.027–2.058), tumor number 2–3 (β = 0.303, 95%CI = 0.916–2.000), tumor number ≥ 4 (β = 1.101, 95%CI = 1.648–5.490), maximal tumor size 3–5 cm (β = 0.553, 95%CI = 1.084–2.787), maximal tumor size > 5 cm (β = 0.516, 95%CI = 1.074–2.613), ES classification III (β = 0.408, 95%CI = 1.059–2.136), presence of microvascular invasion (β = 0.388, 95%CI = 1.022–2.125), AST ≥ 40 (β = 0.690, 95%CI = 1.376–2.887) and GGT ≥ 60 (β = 0.614, 95%CI = 1.273–2.683) were significantly correlated with the shorter OS (*p* < 0.05). Meanwhile, the larger tumor number and larger maximal tumor size with higher β represented greater correlation (Table 5). Therefore, the PS score, tumor number, maximal tumor size, ES classification, microvascular invasion, AST and GGT were the significant independent risk factors of OS, while the combination with TACE/RFA, satellite nodule, TNM staging, AFP and PAB were the risk factors without significance. The OS curve of each factor was shown in Figure 3 (A‐G).

**Table 5:** Multivariable Analysis (Cox Regression) of Risk Factors for OS.

Variables	β	S.E.	Wald	*p*‐value	HR	95%CI
PS score						
0	*Reference*					
≥ 1	0.374	0.177	4.450	0.035*	1.454	1.027–2.058
Tumor number			13.719	0.001*		
1	*Reference*					
2–3	0.303	0.199	3.908	0.044*	1.354	0.916–2.000
≥ 4	1.101	0.307	12.865	< 0.001*	3.008	1.648–5.490
Maximal tumor size			6.515	0.038*		
≤ 3 cm	*Reference*					
3–5 cm	0.553	0.241	5.273	0.022*	1.738	1.084–2.787
> 5 cm	0.516	0.227	5.172	0.023*	1.675	1.074–2.613
TACE/RFA						
No	*Reference*					
Yes	0.284	0.271	3.047	0.125	0.911	1.252–1.774
ES classification						
I–II	*Reference*					
III	0.408	0.179	5.202	0.023*	1.504	1.059–2.136
Microvascular invasion						
Absent	*Reference*					
Present	0.388	0.187	4.310	0.038*	1.474	1.022–2.125
Satellite nodule						
Absent	*Reference*					
Present	0.263	0.190	3.428	0.096	1.391	1.357–2.429
TNM staging			4.290	0.141		
IA	*Reference*					
IB	0.192	0.205	2.681	0.172	1.426	0.924–1.730
II	0.228	0.216	3.105	0.140	1.927	1.163–2.039
IIIA	0.294	0.307	3.793	0.108	1.554	1.715–2.596
AFP						
< 400	*Reference*					
≥ 400	0.109	0.184	1.875	0.126	2.054	1.432–2.157
PAB						
< 80	*Reference*					
≥ 80	0.241	0.293	2.230	0.099	1.636	0.928–1.842
AST						
< 40	*Reference*					
≥ 40	0.690	0.189	13.317	< 0.001*	1.993	1.376–2.887
GGT						
< 60	*Reference*					
≥ 60	0.614	0.190	10.440	0.001*	1.848	1.273–2.683

Abbreviations: AFP, alpha fetoprotein; AST, aspartate aminotransferase; ES, Edmond‐Steiner; GGT, γ‐glutamyl transpeptidase; PAB, prealbumin; PS, performance status; TACE/RFA, transcatheter arterial chemoembolization/radio frequency ablation.

* The factor has statistical significance to OS

## 4 Discussion

The key findings showed that the 1‐, 3‐, and 5‐year DFS rates of patients with HCC after radical operation were 70.67%, 49.65% and 40.65%, while the 1‐, 3‐, and 5‐year OS rates were 90.30%, 79.68% and 70.67%, respectively. In addition, tumor number, microvascular invasion, thickness of resection margin, AFP, AST and GGT were the independent risk factors of DFS, while PS score, tumor number, maximal tumor size, ES classification, microvascular invasion, AST and GGT were the independent risk factors of OS. Li et al. [12] conducted a retrospective analysis of 549 cases after radical resection, and found that the 1‐, 3‐, and 5‐year OS rate were 83.8%, 69.0% and 54.2%, while the 1‐, 3‐, and 5‐year DFS were 61.0%, 44.2% and 36.0%. The recurrence rate after hepatectomy is relatively high, which has a serious negative influence on the life quality of the patient [13], making the curative effect after hepatectomy still face a bottleneck.

In recent years, with the support of increasing evidence‐based evidence, RFA has become another HCC radical operations recommended by the guideline [5]. RFA has the advantages of less trauma and less liver dysfunction. It is pointed out [5] that for patients with early HCC, RFA has similar or slightly lower curative effect than liver resection. A meta‐analysis of 22 studies including 2457 cases by Feng et al. [14] indicated that hepatectomy and RFA had similar short‐term (1‐year) curative effect, while for the long‐term (3–5 years) DFS and OS rates, hepatectomy was superior to RFA. However, RFA could reduce complications and shorten hospital stay. It should be noted that RFA will inevitably damage the liver while destroying tumor cells, and the postoperative local inflammatory reaction will blur the tumor invasion boundary [15], which also lays a hidden danger for recurrence. Whether hepatectomy or RFA, radical operations have limitation on patient′s survival rate. Therefore, The importance of studying the risk factors after radical operation is self‐evident.

The present study showed that the DFS and OS rates at each time point were all higher than those of the previous reports [12, 16, 17]. Based on the analysis of the general clinical data, pathology report and laboratory indicators, we believe that the result may be associated with the relatively better overall condition of the patients in this study.

The current multivariable Cox′s regression analysis showed that the independent prognostic factors of death (DFS/OS) in patients with HCC after radical operation were PS score, tumor number, maximal tumor size, ES classification, microvascular invasion, thickness of resection margin, AFP, AST and GGT. However, thickness of resection margin and AFP were correlated with DFS rather than OS, while PS score, maximal tumor size and ES classification were correlated with OS rather than DFS. The reason is mainly related to the properties of these variables. In most literature, AFP is closely linked to the recurrence of liver cancer, which makes it more relevant to DFS. Meguro et al. [18] analyzed the prognosis of HCC patients infected with hepatitis virus, and discovered that high level of AFP was an independent risk factor associated with tumor recurrence. Sharma et al. [19] pointed out that the serum AFP level per total tumor volume (AFP/TTV) was a better prognostic indicator than that of AFP alone, and the ratio greater than 20 could predict the tumor recurrence. As is known to all, thickness of resection margin refers to the distance between the edge of tumor and the edge of resected tissue. Theoretically, the smaller thickness of resection margin means the closer distance between the tumor and the normal tissue, which will cause higher risk of recurrence. Therefore, larger thickness of resection margin was a significant prognostic factor for DFS [16]. This study came to the same point. Nevertheless, a survival analysis of 1002 patients suggested that there was no correlation between resection margin and DFS [20]. It may be because of the relatively poor condition of the patients (47.8% MELD score > 8) in that study which could increase the risk of recurrence. PS score, as a basic standard of the performance status, is the most common assessment methods of cancer patients. Some studies show that PS score is closely related to both DFS and OS [21, 22], which is inconsistent with our result. PS score mainly represents patients′ overall condition rather than the probability of recurrence. We believe that′s why PS score only has correlation with OS in the present study. It is worth mentioning that maximal tumor size and ES classification are correlated with only OS. Theoretically, these two variables belong to the properties of tumor, which will not only affect the recurrence, but also have influence on the patients′ overall condition (larger tumor tends to consume more nutrients, and higher ES grade means higher degree of malignancy and more serious condition). In addition, this study showed that age, TNM staging and hepatitis were not significant risk factors. On the one hand, it may be related to the early clinical stage (85.68% stage I‐II) which can decrease the risk of death. On the other hand, the regional and ethnic difference of the subjects is also a possible reason.

In general, the concluded prognostic factors of DFS or OS are consistent with previous studies [23, 24, 25, 26]. Among these, tumor number, maximal tumor size and microvascular invasion are the major components of the American Joint Committee on Cancer (AJCC) 8th staging system [10]. In addition, PS score, ES classification, thickness of resection margin, AFP, AST and GGT have also been proved to be relevant factors. However, our study indicated that age, hepatitis, TNM staging and vascular invasion were not risk factors, which was inconsistent with most other studies. Moreover, the correlations between each factor and DFS or OS were also different. These points are still controversial, thus, our results needs further research.

The current study has several limitations. First, the sample size was relatively small (largely due to the recent implementation of an updated medical record system). Increasing the sample size in future research would help achieve a more balanced distribution across clinical stages, minimize data bias, reduce potential errors, and possibly lead to more conclusive or even transformative findings. Second, all participants were recruited from a single center, which restricts the generalizability of the results. Thus, future clinical studies should aim to be multicenter in design, incorporate larger samples, and maintain high methodological quality to provide physicians reliable evidence‐based guidance.

## 5 Conclusion

In summary, the independent risk factors for mortality in HCC patients following radical resection include PS score, tumor number, maximal tumor size, ES classification, microvascular invasion, resection margin thickness, AFP, AST, and GGT. Among these, tumor number, microvascular invasion, AST, and GGT are associated with both DFS and OS, indicating their potential utility as predictors for postoperative prognosis and recurrence in HCC. Timely identification of patients with these risk factors is clinically important, as it may help prevent tumor‐related events and improve survival outcomes.

## Author Contributions

Zhengyang He and Dongze Qiu are co‐first authors of this manuscript. Zhengyang He designed the study and collected the data. Dongze Qiu did the data analysis. Zhengyang He wrote the manuscript. Weimin She revised the manuscript and decided to submit the manuscript for publication. All authors read and approved the final manuscript.

## Conflicts of Interest

All authors read and approved the final manuscript and declare that they have no competing interests.

## Transparency Statement

The lead author Dongze Qiu, Weimin She affirms that this manuscript is an honest, accurate, and transparent account of the study being reported; that no important aspects of the study have been omitted; and that any discrepancies from the study as planned (and, if relevant, registered) have been explained.

## Data Availability

All supporting data can be provided upon request to the authors. All authors have read and approved the final version of the manuscript. QDZ and SWM had full access to all of the data in this study and takes complete responsibility for the integrity of the data and the accuracy of the data analysis.
